# Pandemic-Associated Disruption and Regional Divergence in Childhood Obesity Trajectories in Brazil, 2008-2025

**DOI:** 10.7759/cureus.117593

**Published:** 2026-10-07

**Authors:** Carolina O Nanni, Marcos R Nanni, Isadora O Nanni, Cintia Motta, Ricardo Fava, Kézia de Oliveira, Ana Carolina Santana da Silva Mangialardo, Nádia Sogabe, Jácomo Manzotti, Eduardo Moreschi

**Affiliations:** 1 Department of Medicine, Centro Universitário Cesumar, Maringá, BRA; 2 Department of Agronomy, Universidade Estadual de Maringá, Maringá, BRA; 3 Department of Medicine, Universidade Estadual de Londrina, Londrina, BRA; 4 Department of Internal Medicine, Universidade Nove de Julho, Maringá, BRA; 5 Department of Medicine, Centro Universitário Ingá, Maringá, BRA

**Keywords:** brazil, childhood obesity, covid-19, nutritional surveillance, pediatric-onset obesity

## Abstract

Background: National averages may conceal whether Brazilian regions experienced different departures from, and returns to, their projected pre-pandemic childhood obesity trajectories. We evaluated long-term trends, pandemic-associated deviations, and changes in regional inequality among children aged 5-10 years monitored by the Brazilian Food and Nutrition Surveillance System (SISVAN) from 2008 through 2025.

Methods: We conducted a nationwide ecological interrupted time-series study using publicly available surveillance data. Joinpoint regression estimated annual percent changes. Segmented regression estimated the 2020 level change and subsequent slope change nationally and in each region. From these coefficients, we calculated annual and cumulative fitted deviations from continuation of the pre-pandemic trajectory. Regional absolute and relative gaps were assessed at prespecified years.

Results: Prevalence increased from 10.45% in 2008 to 15.41% in 2025 and peaked at 17.84% in 2021. The national model showed a 2.54-percentage-point level increase in 2020 (P=0.001) and a -0.646-percentage-point annual slope change thereafter (P=0.002). The fitted deviation from the projected pre-pandemic trajectory decreased from +2.54 points in 2020 to approximately zero in 2024 and -0.69 in 2025. Regional recovery diverged: the South and Southeast crossed their projected trajectories in 2023, the Northeast and Center-West in 2025, whereas the North remained 1.28 points above projection in 2025. The maximum regional prevalence gap widened from 3.18 points in 2008 to 10.78 in 2021, then narrowed to 6.66 in 2025.

Conclusions: The pandemic period was associated with a transient national departure from the pre-pandemic trajectory, but recovery was regionally asynchronous. Persistent excess relative to projection in the North contrasted with faster reversal in the South and Southeast, while regional inequality remained wider in 2025 than in 2008. Interpretation must account for changes in surveillance coverage.

## Introduction

Childhood obesity is a major global public health challenge, with prevalence increasing substantially over recent decades because of complex interactions among behavioral, environmental, and socioeconomic factors [1,2]. Childhood obesity is associated with type 2 diabetes, cardiovascular disease, metabolic syndrome, and persistence of obesity into adulthood [1]. However, its distribution varies across regions and populations because of socioeconomic inequalities, differences in healthcare access, dietary patterns, and environmental exposures [3-5].

The COVID-19 pandemic disrupted children’s daily routines through school closures, reduced access to recreational spaces, and social distancing. These changes contributed to decreased physical activity, increased sedentary behavior, and altered dietary habits, and studies reported increases in childhood obesity during the pandemic [6-10]. In Brazil, the Food and Nutrition Surveillance System (SISVAN) provides nationwide longitudinal surveillance of children receiving care through the public health system [11]. Evidence from pediatric studies and reviews also indicates increased weight gain and obesity incidence during and after the pandemic period [12-14]. These changes occurred against a broader global trend of increasing obesity prevalence [15], while modeling studies suggest that pandemic-associated increases in childhood obesity may produce substantial long-term health and economic consequences [16]. Previous reports have described temporal trends in childhood excess weight in Brazil [17], but a clinically and epidemiologically distinct question remains: whether the 2020 disruption produced a temporary or persistent departure from the trajectory already underway, whether regions returned to that projected course at different times, and whether between-region inequality widened during this process.

This study aimed to quantify pandemic-associated departures from the projected pre-pandemic trajectory of childhood obesity prevalence among Brazilian children aged 5-10 years from 2008 through 2025. We also assessed the timing and magnitude of regional return toward projected trajectories and changes in absolute and relative inequality across Brazil’s five geographic regions.

## Materials and methods

Study design and data source

We conducted a nationwide ecological interrupted time-series study to evaluate temporal trends in childhood obesity in Brazil from 2008 through 2025. We obtained publicly available data from the SISVAN, maintained by the Brazilian Ministry of Health [11]. SISVAN monitors anthropometric and nutritional indicators among population groups receiving care through the Brazilian public health system.

We obtained data from annual consolidated reports available through the SISVAN public consultation platform. We collected information separately for each year at the national level and for Brazil’s five geographic regions: North, Northeast, Southeast, South, and Center-West.

Study population

The study population comprised Brazilian children aged 5-10 years evaluated by SISVAN from 2008 through 2025. We conducted analyses at the national level and separately for each geographic region.

Data extraction and selection criteria

We manually extracted data from the annual SISVAN reports by using standardized selection criteria to ensure comparability across the study period. We applied the following filters: children aged 5-10 years; body mass index (BMI)-for-age as the anthropometric indicator; obesity as the nutritional classification; and the World Health Organization (WHO) 2007 growth reference for children and adolescents aged 5-19 years [12].

We included all complete annual datasets available for the study period; therefore, sampling and a pre-study sample-size calculation were not applicable.

Variables and definitions

In accordance with the WHO 2007 growth reference, we defined childhood obesity as BMI-for-age >2 standard deviations above the reference median [12]. The primary outcome was annual national obesity prevalence, expressed as the percentage of evaluated children classified as having obesity.

Secondary outcomes included regional-level and slope changes; fitted annual and cumulative deviations from continuation of the pre-pandemic trajectory; the year in which each fitted regional trajectory first reached or crossed its counterfactual projection; and absolute and relative regional inequality at prespecified years (2008, 2019, 2021, and 2025).

Statistical analysis

We performed descriptive analyses of annual childhood obesity prevalence at the national and regional levels. We used the Joinpoint Regression Program version 6.0.0 (National Cancer Institute, Bethesda, USA) [18] to estimate annual percent changes (APCs) and corresponding 95% CIs [15]. We fitted log-linear models to identify changes in temporal trends and allowed a maximum of 1 joinpoint because the series contained 18 annual observations.

We used Stata/MP, version 14.0 (StataCorp LLC, College Station, USA), to perform interrupted time-series analyses of level and slope changes associated with the COVID-19 period [19]. We designated 2020 as the interruption point and fitted segmented linear regression models according to the following equation: 

Y_t_ = β_0_ + β_1_(time_t_) + β_2_(COVID-19 period_t_)+ β_3_(post-interruption time_t_)+ ε_t_

In this equation, Y_t_ represents childhood obesity prevalence in year t. We coded time sequentially from 1 in 2008 to 18 in 2025. We coded the COVID-19 period indicator as 0 from 2008 through 2019 and 1 from 2020 through 2025. We coded post-interruption time as 0 through 2020 and sequentially from 1 in 2021 to 5 in 2025. Therefore, β_1_ represents the annual pre-pandemic trend, β_2_ represents the immediate level change in 2020, and β_3_ represents the change in slope after the interruption.

We first fitted a national model using annual overall obesity prevalence and then fitted separate models for each geographic region to assess regional heterogeneity. We assessed first-order residual autocorrelation in the national model using the Durbin-Watson statistic. Because SISVAN surveillance coverage varied over time and declined substantially in 2020, we interpreted estimates associated with the COVID-19 period cautiously. We used two-sided tests and defined statistical significance as P<0.05.

For each year from 2020 through 2025, we derived the fitted deviation from continuation of the pre-pandemic trajectory as β_2_ + β_3_k, where k equaled 0 in 2020 and 1 through 5 in 2021 through 2025. We summed these annual deviations to describe cumulative deviation in percentage-point-years. These derived estimates are descriptive functions of the segmented models and were not interpreted as causal effects. We also calculated the absolute regional gap as the difference between the highest and lowest regional prevalence and the relative gap as their ratio.

## Results

We analyzed 18 years of nationwide surveillance data from 2008 through 2025. Childhood obesity prevalence increased from 10.45% in 2008 to 15.41% in 2025. Prevalence increased steadily from 10.45% in 2008 to 13.33% in 2017 and remained near 13% from 2017 through 2019. It then increased from 13.19% in 2019 to 15.84% in 2020 and peaked at 17.84% in 2021. Prevalence subsequently declined to 15.83% in 2022 and 14.36% in 2023 before increasing slightly to 15.41% in 2025.

Interrupted time-series regression showed a significant pre-pandemic increase of 0.268 percentage points per year (β=0.268; 95% CI, 0.148 to 0.388; P<0.001). The COVID-19 period was associated with an immediate level increase of 2.54 percentage points in 2020 (β=2.54; 95% CI, 1.17 to 3.91; P=0.001). The post-interruption slope change was negative (β=-0.646 percentage points per year; 95% CI, -1.010 to -0.281; P=0.002). The model explained 90.1% of the variation in obesity prevalence (R^2^=0.901). The Durbin-Watson statistic was 1.91, indicating no substantial first-order residual autocorrelation (Figure 1).

**Figure 1:**
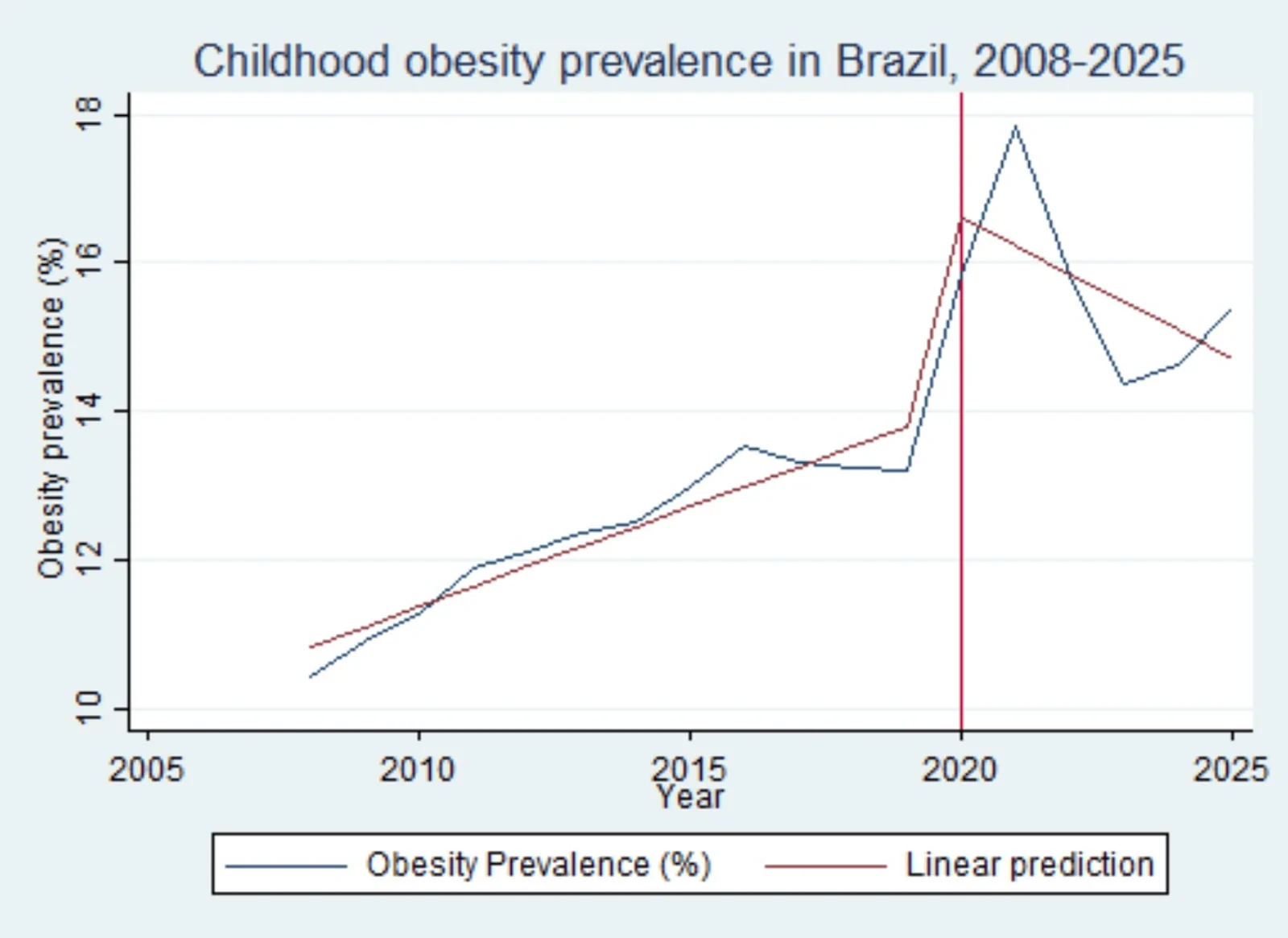
Interrupted time-series analysis of childhood obesity prevalence among children aged 5-10 years monitored by SISVAN, Brazil, 2008-2025. The blue line represents observed annual obesity prevalence, and the red line represents values predicted by segmented linear regression. The vertical red line indicates the interruption point in 2020, corresponding to the beginning of the COVID-19 period. SISVAN: Food and Nutrition Surveillance System

Joinpoint regression identified a significant increase in childhood obesity prevalence from 2008 through 2021 (APC=3.09%; 95% CI, 2.35% to 6.70%; P=0.015). From 2021 through 2025, prevalence showed a nonsignificant decreasing trend (APC=-1.35%; 95% CI, -8.42% to 2.06%; P=0.47) (Table 1 and Figure 2).

**Table 1:** Joinpoint regression analysis of temporal trends in childhood obesity in Brazil (2008-2025). Annual percent change (APC), 95% CI, and p-values for temporal trends in childhood obesity prevalence among Brazilian children aged 5-10 years.

Segment	Lower endpoint	Upper endpoint	APC (%)	95% CI	P-value
1	2008	2021	3.09	2.35 to 6.70	0.015
2	2021	2025	-1.35	-8.42 to 2.06	0.47

**Figure 2:**
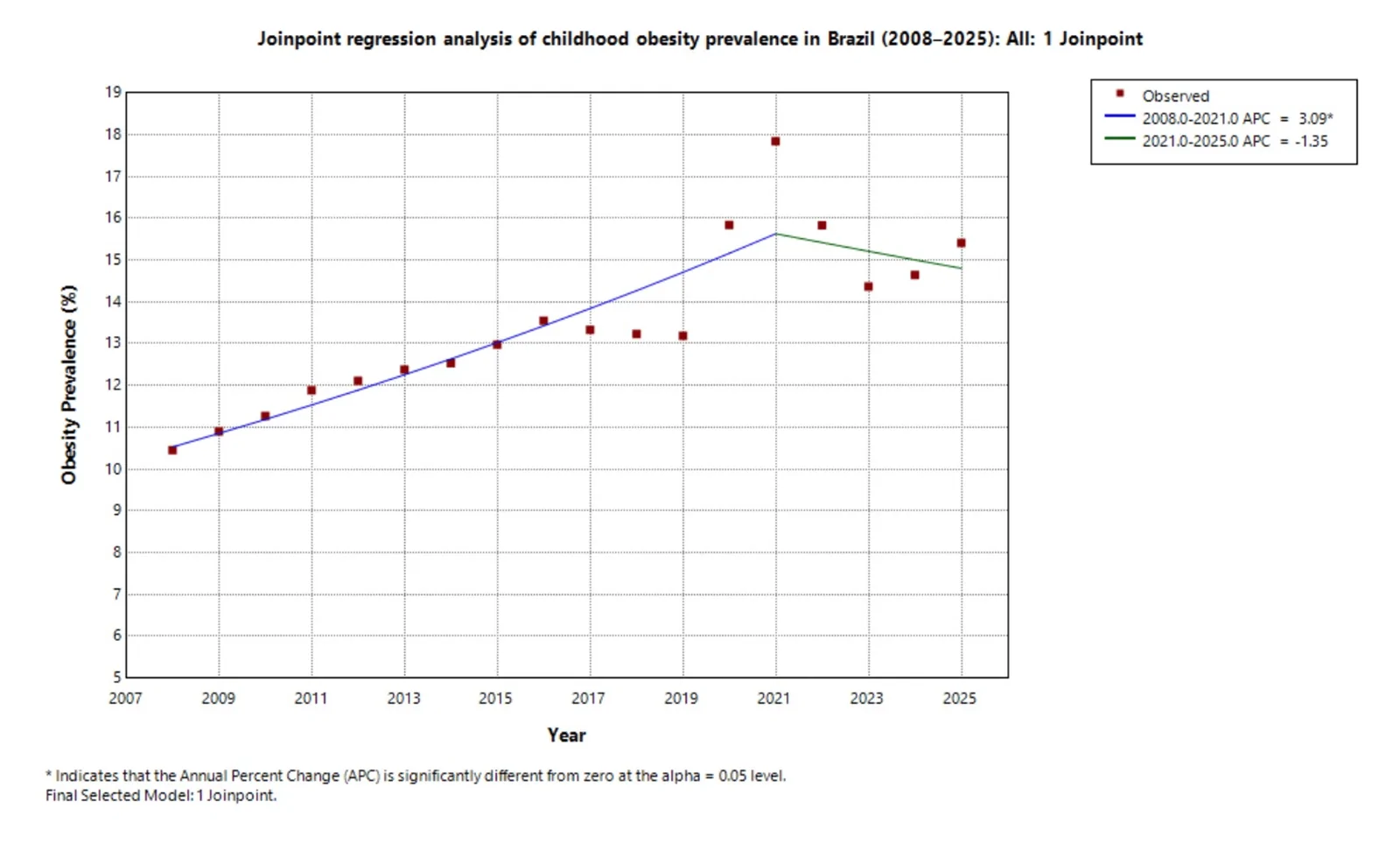
Temporal trends in childhood obesity prevalence among Brazilian children aged 5-10 years according to Joinpoint regression analysis.

Regional trends

Obesity prevalence differed across Brazil’s geographic regions throughout the study period (Table 2). The South and Southeast consistently had the highest prevalence, whereas the North had the lowest. In 2008, prevalence ranged from 8.24% in the North to 11.42% in the South.

**Table 2:** Regional changes in childhood obesity prevalence in Brazil (2008-2025). Childhood obesity prevalence (%) across Brazilian regions during pre-pandemic, pandemic, and post-pandemic periods.

Region	2008 (%)	2019 (%)	2021 (%)	2025 (%)	Absolute change (2008-2025)	Pandemic increase (2019-2021)	Post-pandemic change (2021-2025)
North	8.24	8.45	11.85	10.76	+2.52	+3.40	-1.09
Northeast	10.76	13.37	17.32	15.87	+5.11	+3.95	-1.45
Southeast	10.58	14.48	19.89	16.48	+5.90	+5.41	-3.41
South	11.42	15.97	22.63	17.42	+6.00	+6.66	-5.21
Center-West	10.41	12.41	16.74	13.76	+3.35	+4.33	-2.98

All regions had higher prevalence in 2025 than in 2008. From 2019 through 2021, the South had the largest absolute increase, from 15.97% to 22.63% (+6.66 percentage points), followed by the Southeast, from 14.48% to 19.89% (+5.41 percentage points). The North had the smallest increase, from 8.45% to 11.85% (+3.40 percentage points). Prevalence declined in all regions from 2021 through 2025 but remained above 2019 levels. In 2025, prevalence was highest in the South (17.42%) and Southeast (16.48%) and lowest in the North (10.76%).

Regional interrupted time-series models showed heterogeneous temporal patterns (Table 3). The South had the steepest pre-pandemic increase (β=0.466 percentage points per year; 95% CI, 0.289 to 0.642; P<0.001), followed by the Southeast (β=0.385; 95% CI, 0.229 to 0.540; P<0.001). The pre-pandemic trend in the North was smaller and not significant (β=0.058; 95% CI, -0.039 to 0.154; P=0.221).

**Table 3:** Regional interrupted time-series regression analysis of childhood obesity prevalence in Brazil (2008-2025). Interrupted time-series regression coefficients evaluating temporal changes in childhood obesity prevalence across Brazilian regions during the COVID-19 period.

Region	Pre-pandemic trend β (95% CI)	P	Immediate level change β (95% CI)	P	Post-interruption slope change β (95% CI)	P	R^2^
North	0.058 (-0.039 to 0.154)	0.221	2.189 (1.095 to 3.283)	<0.001	-0.182 (-0.473 to 0.110)	0.203	0.839
Northeast	0.260 (0.152 to 0.368)	<0.001	1.882 (0.653 to 3.111)	0.005	-0.408 (-0.736 to -0.081)	0.018	0.909
Southeast	0.385 (0.229 to 0.540)	<0.001	2.810 (1.043 to 4.576)	0.004	-1.025 (-1.496 to -0.554)	<0.001	0.888
South	0.466 (0.289 to 0.642)	<0.001	3.639 (1.633 to 5.646)	0.002	-1.448 (-1.983 to -0.914)	<0.001	0.898
Center-West	0.152 (0.042 to 0.263)	0.010	3.037 (1.787 to 4.288)	<0.001	-0.750 (-1.084 to -0.417)	<0.001	0.870

The South had the largest immediate level change in 2020 (β=3.639 percentage points; 95% CI, 1.633 to 5.646; P=0.002), followed by the Center-West (β=3.037; 95% CI, 1.787 to 4.288; P<0.001) and Southeast (β=2.810; 95% CI, 1.043 to 4.576; P=0.004). Significant negative post-interruption slope changes occurred in the Northeast, Southeast, South, and Center-West but not in the North.

Derived deviations from the projected pre-pandemic trajectories further distinguished the regional recovery patterns. Nationally, the fitted deviation decreased from +2.54 percentage points in 2020 to +0.60 in 2023, approximately zero (-0.04) in 2024, and -0.69 in 2025; the cumulative deviation for 2020-2025 was +5.55 percentage-point-years. The South and Southeast crossed below their projected pre-pandemic trajectories in 2023, the Northeast and Center-West did so in 2025, and the North did not return to projection during follow-up, remaining +1.28 percentage points above it in 2025. Cumulative deviation was greatest in the North (+10.40 percentage-point-years), followed by the Center-West (+6.97) and Northeast (+5.17).

Between-region inequality widened during the study. The absolute gap between the regions with the highest and lowest prevalence increased from 3.18 percentage points in 2008 to 7.52 in 2019 and 10.78 in 2021, before narrowing to 6.66 in 2025. Corresponding highest-to-lowest prevalence ratios were 1.39, 1.89, 1.91, and 1.62, respectively. Thus, both absolute and relative inequality remained greater in 2025 than at the beginning of the series.

Surveillance coverage

The number of children evaluated by SISVAN varied across regions and years. Surveillance coverage declined markedly in 2020 and progressively recovered thereafter. In the Northeast, for example, the number of evaluated children decreased from approximately 1.84 million in 2019 to 880,089 in 2020 and subsequently increased to more than 2.1 million in 2025.

## Discussion

This analysis moves beyond documenting an updated national trend by estimating how far observed fitted trajectories departed from the continuation of their pre-pandemic course, when they returned toward that course, and how regional inequality changed during the disruption. Three findings stand out. First, the national pandemic-associated deviation was transient in the segmented model, approaching zero by 2024 despite prevalence remaining above its 2019 value. Second, recovery was asynchronous: the South and Southeast reversed most rapidly, whereas the North retained a positive deviation through 2025. Third, the gap between the highest- and lowest-prevalence regions widened markedly through 2021 and remained larger in 2025 than in 2008. These findings distinguish persistence above a historical baseline from persistence above an expected trajectory-two interpretations with different implications for surveillance and intervention.

Joinpoint and interrupted time-series analyses answered complementary questions. Joinpoint identified 2021 as the data-driven change from sustained increase to nonsignificant decline. Segmented regression estimated the departure associated with the prespecified 2020 interruption after accounting for the prior slope. The counterfactual comparison shows why using 2019 alone as the benchmark can be misleading: because obesity was already increasing, prevalence could remain above 2019 even after returning to, or falling below, its projected pre-pandemic trajectory. These estimates describe model-based deviations associated with calendar time and should not be interpreted as causal effects of the pandemic.

The South and Southeast showed the clearest pattern of cumulative and persistent high burden. Both regions had relatively high prevalence at the beginning of the series, steep pre-pandemic increases, and large absolute increases between 2019 and 2021. The South had the steepest pre-pandemic slope, the largest immediate level change, and the highest prevalence in both 2021 and 2025. Although it also had the strongest negative post-interruption slope, this decline began from the highest peak and was insufficient to erase the accumulated increase. The Southeast followed a similar pattern, with a pronounced pre-pandemic increase, a substantial 2020 level change, and a significant subsequent decline, while remaining the region with the second-highest prevalence in 2025. These findings show why a negative post-2020 slope should not be interpreted as resolution: rapid improvement after an exceptional peak can coexist with a persistently high absolute burden.

The Center-West displayed a different profile. Its pre-pandemic slope was considerably smaller than those of the South and Southeast, yet its immediate level increase in 2020 was the second largest among the regions. This contrast suggests a sharper temporal disruption relative to its previous trajectory rather than simple acceleration of an already steep trend. The Northeast, in turn, combined a significant pre-pandemic increase with a smaller immediate level change and a significant negative post-interruption slope. Despite that decline, its net increase from 2008 to 2025 was exceeded only by those in the South and Southeast, indicating that the longer-term accumulation of obesity may be more epidemiologically important than the isolated pandemic peak.

The North had the lowest measured prevalence throughout the study but the most persistent positive deviation from its projected pre-pandemic trajectory. Unlike the other regions, it did not cross back to projection by 2025 and accumulated the largest model-derived deviation over 2020-2025. This finding shifts interpretation away from a simple ranking based on absolute prevalence: a region can have a lower measured burden yet experience a more persistent disruption relative to its own historical course. Greater heterogeneity among states and municipalities, unequal access to primary care, and changes in the population captured by surveillance remain plausible alternative explanations.

Recent Brazilian evidence supports the interpretation that nutritional transition is spatially heterogeneous. An analysis of children aged 5-9 years registered in SISVAN from 2008 to 2022 found increasing overweight in both semi-arid and non-semi-arid areas, with a greater annual increase in the semi-arid region and persistent coexistence of overweight and undernutrition [17]. Therefore, the higher absolute obesity prevalence observed in the South and Southeast should not be interpreted as a simple inverse socioeconomic gradient. Regions with lower measured obesity may simultaneously experience rapid nutritional transition, food insecurity, undernutrition, and unequal access to preventive care. The regional pattern is better understood as the product of different stages and expressions of nutritional transition than as a ranking from more to less affected.

Several pathways may have contributed to the abrupt changes observed during the COVID-19 period. School closures reduced opportunities for structured physical activity and access to school meals; restrictions altered sleep, screen exposure, family routines, and food purchasing; and economic instability may have increased reliance on inexpensive energy-dense foods [6-14]. A review of post-COVID-19 syndrome in children also discusses school closure and social isolation as social factors potentially affecting children’s behavior [20]. However, the present data contain no individual measures of diet, physical activity, income, schooling, or household conditions. These mechanisms are therefore plausible contextual explanations rather than tested mediators. Regional differences in restrictions, school reopening, urbanization, food environments, and social protection may have influenced the magnitude of change, but the ecological design does not allow their independent contributions to be estimated.

Interpretation of the 2020 level change must also account for surveillance dynamics. SISVAN represents children monitored in primary care and social programs rather than a probabilistic sample of all Brazilian children. Previous evaluations have shown expansion of the system alongside persistent geographic variation in coverage and have emphasized that changes in the composition of monitored children can affect temporal comparisons [21]. In the present series, the number of evaluated children fell sharply in 2020 and recovered thereafter. If access to routine care declined selectively, the children measured during the most disrupted period may have differed from those measured before or after it. The estimated level change may consequently reflect both a true change in obesity prevalence and a change in who entered the surveillance denominator. Because coverage and access differ regionally, this potential selection process may also have contributed to the heterogeneous coefficients.

These limitations do not negate the observed trends, but they define their appropriate use. SISVAN provides a uniquely long and operationally relevant view of children receiving care in the Brazilian Unified Health System. Its anthropometric database has high completeness for core measurements, although evaluations of child data have identified variation in coverage and data quality over time [22]. The consistency between the national Joinpoint and interrupted time-series results, the persistence of prevalence above 2019 levels in every region, and the coherent regional patterns across several indicators support the conclusion that childhood obesity remains a substantial problem. At the same time, prevalence estimates should not be generalized uncritically to all Brazilian children or interpreted as direct measures of pandemic causation.

The findings have practical implications for pediatric care and public health. In the South and Southeast, where high baseline prevalence and large pandemic-associated increases converged, priorities include systematic BMI-for-age monitoring, early identification of rapid weight gain, and sustained multidisciplinary management in primary care. In the Center-West, the pronounced immediate shift supports attention to service and school disruptions that may have rapidly altered children’s routines. In the Northeast and North, strategies must avoid focusing on obesity in isolation: prevention should be integrated with food-security policies and surveillance of all forms of malnutrition. Across regions, school-based physical activity and nutrition programs, continuity of anthropometric monitoring, family-centered counseling, and referral pathways for children with severe obesity or comorbidities should be strengthened. Regional tailoring should complement, not replace, universal national policies.

This study has important strengths. It analyzed 18 consecutive years, combined data-driven and prespecified interrupted-trend methods, modeled each region separately, and distinguished comparison with a fixed 2019 baseline from comparison with the projected pre-pandemic trajectory. Derived deviation and inequality measures made the timing and heterogeneity of recovery explicit. Limitations include the ecological design, lack of individual-level covariates, inability to distinguish repeated from newly observed children, absence of a concurrent control series, potential changes in measurement and reporting practices, and regional variation in surveillance coverage. The derived counterfactuals depend on linear continuation of the pre-pandemic trend and do not establish pandemic causality. Uncertainty intervals for derived deviations were not estimated because the covariance between level- and slope-change coefficients was unavailable in the aggregated output. Analyses were not stratified by sex, narrower age groups, race or ethnicity, municipality, or socioeconomic position.

## Conclusions

In conclusion, the 2020 disruption was associated with a transient departure from the projected national childhood obesity trajectory, but the return was asynchronous across regions. South and Southeast crossed their projected trajectories by 2023 after the largest initial increases, whereas the North retained a positive deviation through 2025 and accumulated the greatest model-derived excess relative to its previous course. Regional inequality narrowed after the 2021 peak but remained wider than in 2008. Surveillance and prevention strategies should therefore distinguish high absolute prevalence from persistent deviation relative to regional history and should explicitly account for changes in SISVAN coverage.
